# Primary Radiotherapy Versus Surgery in Early-Stage Endometrial Cancer Among High-Risk Surgical Patients: A Retrospective Comparative Study

**DOI:** 10.3390/cancers18111858

**Published:** 2026-06-05

**Authors:** Lucia Gómez-Lavin Fernández, Sergi Fernández-González, Dina Najjari-Jamal, Lola Marti Cardona, Marc Juarez Lozano, Marc Barahona, Marta Gil Martin, Beatriz Pardo Burdalo, Andrea Slocker Escarpa, Milica Stefanovic, Cristina Gutiérrez Miguelez, Jordi Ponce Sebastià

**Affiliations:** 1 Department of Obstetrics and Gynecology, Parc Taulí Hospital Universitari, Institut d’Investigació i Innovació Parc Taulí (I3PT-CERCA), Universitat Autònoma de Barcelona, 08202 Sabadell, Barcelona, Spain; lgomezlavin@tauli.cat; 2 Department of Gynecology, Hospital de Bellvitge, University of Barcelona, 08907 L’Hospitalet de Llobregat, Barcelona, Spain; sfernandez@bellvitgehospital.cat (S.F.-G.); lmarti@bellvitgehsopital.cat (L.M.C.); jponce@bellvitgehospital.cat (J.P.S.); 3 Department of Radiation Oncology, Catalan Institute of Oncology, Campus Salut Bellvitge—Comprehensive Cancer Center, 08908 L’Hospitalet de Llobregat, Barcelona, Spain; mjuarez@iconcologia.net (M.J.L.);; 4 Department of Medical Oncology, Catalan Institute of Oncology, Campus Salut Bellvitge—Comprehensive Cancer Center, 08908 L’Hospitalet de Llobregat, Barcelona, Spain; 5 Department of Radiation Oncology, Hospital del Mar, 08003 Barcelona, Barcelona, Spain

**Keywords:** endometrial cancer, brachytherapy, external beam radiotherapy, morbidity, morbid obesity

## Abstract

**Background/Objectives:** Surgery is the standard treatment for early-stage endometrial cancer (EC); however, some patients are medically inoperable due to comorbidities or morbid obesity. Definitive image-guided brachytherapy (BT) with or without external beam radiotherapy (EBRT) is a curative alternative, although comparative data with surgery remains limited. This study compared cancer-specific survival (CSS) at 2 and 5 years in high-risk surgical patients with early-stage EC (FIGO 2009 I–II), treated with definitive radiotherapy or surgery. Secondary endpoints included overall survival (OS), recurrence-free survival (RFS), disease-free survival (DFS), and toxicity. **Methods:** Retrospective comparative study including 72 patients treated between 2011 and 2023: 36 medically inoperable treated with BT +/− EBRT and 36 matched undergoing surgery. Survival outcomes were estimated using Kaplan–Meier methods and compared using log-rank tests. **Results:** Median age was 74 years and mean BMI was 38.1 kg/m^2^; 75% were morbidly obese, with endometrioid carcinoma being the predominant histology (88.9%). Five-year CSS was 97.2% in the radiotherapy group versus 91.7% in the surgery group (*p* = 0.39). Five-year RFS was identical in both groups (86.1%), with recurrence rates of 13.9%. Five-year OS was lower in the radiotherapy group (66.7% vs. 77.8%; *p* = 0.3), without statistical significance. Grade ≥3 radiotherapy-related toxicity occurred in 19.4%, whereas severe surgical complications occurred in 8.3%. **Conclusions:** Definitive BT ± EBRT is an effective and well-tolerated curative option for medically inoperable early-stage EC, with survival and recurrence outcomes comparable to surgery, supporting its role as a valid therapeutic alternative in high-risk surgical patients.

## Simple Summary

A proportion of patients with early-stage endometrial cancer cannot undergo surgery due to severe obesity, advanced age, or other medical conditions. In these cases, radiotherapy may offer a curative alternative, although direct comparisons with surgery remain limited. In this study, we compared two similar groups of patients with early-stage disease (FIGO 2009 stage I–II): one treated with radiotherapy and the other with surgery. Both approaches achieved comparable outcomes in terms of cancer control, survival, and risk of recurrence, with no significant differences in severe treatment-related complications. These findings support radiotherapy as an effective and well-tolerated treatment option for patients who are not suitable for surgery, helping guide treatment decisions in medically high-risk populations.

## 1. Introduction

Endometrial cancer (EC) is the most common gynecologic malignancy in developed countries, and its incidence continues to increase worldwide. This trend is driven by aging populations and the growing prevalence of obesity and metabolic disorders, which are well-established risk factors for EC [1,2]. In Europe, EC accounts for approximately 4.5% of all cancers in women, with an incidence of 10.4 per 100,000 women and a mortality rate of 2.4 per 100,000 in Spain [2,3]. Notably, a growing proportion of patients diagnosed with EC present with severe or extreme obesity, a condition frequently associated with multiple comorbidities such as diabetes, cardiovascular disease, and respiratory impairment. These conditions significantly increase perioperative risk and represent an important challenge for the management of EC.

Surgery remains the standard treatment for early-stage EC and typically consists of total hysterectomy with bilateral salpingo-oophorectomy with or without lymph node assessment [4,5]. In recent years, minimally invasive approaches, particularly robotic-assisted surgery, have expanded surgical eligibility among obese patients by improving visualization, allowing lower intra-abdominal pressures, and facilitating anesthetic management. Robotic surgery has demonstrated advantages over conventional laparoscopy, including reduced blood loss, shorter hospital stays, and lower postoperative complication rates in obese patients [6,7,8]. However, despite these advances, a subset of patients with extreme obesity or severe comorbidities remains medically inoperable because they cannot safely tolerate anaesthesia or surgical positioning. In these situations, clinical guidelines recommend considering definitive radiotherapy (RT) or other non-surgical therapeutic approaches [5].

Definitive RT, particularly brachytherapy (BT) with or without external beam radiotherapy (EBRT), has emerged as a potentially curative alternative for medically inoperable EC [9,10,11]. Advances in three-dimensional image-guided brachytherapy (IGBT) have improved treatment precision by allowing better target coverage and dose escalation while reducing radiation exposure to surrounding organs at risk. Retrospective studies have reported encouraging oncologic outcomes in patients with early-stage EC treated with curative-intent radiotherapy, particularly in terms of local disease control [12,13].

Despite these encouraging results, robust comparative evidence between definitive RT and primary surgery in early-stage EC remains limited. Most available studies are retrospective and lack direct comparisons between treatment modalities, making it difficult to determine whether RT provides oncologic outcomes comparable to high-risk surgical patients. The primary objective of the present study was to compare oncologic outcomes between definitive RT and primary surgery in patients with early-stage EC, with particular emphasis on local disease control. Our hypothesis is that RT provides disease control comparable to surgery in patients with early-stage EC who present with high surgical risk. Accordingly, this study aims to evaluate cancer-specific survival and recurrence outcomes in patients with early-stage endometrial cancer treated with primary surgery or definitive BT ± EBRT.

## 2. Materials and Methods

A retrospective matched cohort study was conducted with high-risk surgical patients diagnosed with endometrial cancer (EC) and treated at University Hospital of Bellvitge (HUB) and Catalan Institute of Oncology (ICO) between September 2011 and May 2023. Eligible patients had a histologically confirmed diagnosis of clinical–radiological FIGO 2009 stage I–II EC and received either primary surgery or definitive radiotherapy (RT) as their initial treatment. Patients with lymph node metastases at diagnosis, distant metastases, prior chemotherapy or RT, or insufficient follow-up data were excluded from the analysis.

We identified 36 patients with clinical–radiological FIGO stage I–II EC treated with definitive RT, consisting of BT with or without EBRT. During the same period, 803 patients underwent primary surgical management. From this cohort, 36 patients were selected and individually matched (1:1) with those in the RT group according to clinical stage, body mass index (BMI), and Charlson comorbidity index (CCI) to ensure comparability between groups. Matching was performed manually on an individual basis by a clinician with expertise in EC management, without the use of computer-assisted algorithms or propensity score matching software. Molecular profile was not included as a matching criterion because comprehensive molecular classification data were unavailable for a substantial proportion of patients in the RT cohort, which would have precluded adequate matching and significantly reduced the eligible sample size. This limitation is acknowledged and its potential impact on between-group comparability is discussed further below.

All cases were discussed on a multidisciplinary tumor board, where surgical risk and the feasibility of curative RT were assessed to determine the optimal treatment strategy. This study received IRB approval from the institution (protocol 180/25), and informed consent was waived, given its retrospective design, in accordance with applicable regulations.

Surgical management consisted of total hysterectomy with bilateral salpingo-oophorectomy. The preferred approach for early-stage EC was minimally invasive surgery, including laparoscopic or robotic-assisted techniques, in accordance with current standards of care. However, all surgical approaches performed during the study period were included in the analysis—minimally invasive (laparoscopic or robotic-assisted), open surgery (laparotomy), and vaginal surgery—depending on clinical indication and patient characteristics. Lymph node assessment was intended as part of the standard surgical management in abdominal procedures and performed whenever feasible according to institutional protocols, including sentinel lymph node biopsy or lymphadenectomy. Adjuvant treatments (BT, EBRT, or chemotherapy) were administered based on established risk factors.

Definitive RT was delivered with curative intent and consisted of BT, either alone or in combination with EBRT, according to tumor characteristics and patient clinical condition. BT was performed using IGBT, with individualized treatment planning based on the residual tumor at time of BT. Following applicator insertion, treatment planning imaging was acquired using computed tomography (CT). For CT-based planning, clinical target volume (CTV) delineation was based on planning CT images in combination with pre-treatment magnetic resonance imaging (MRI) and intraoperative clinical findings, while transrectal ultrasound was used for target volume assessment and measurement. Target definition followed GEC-ESTRO (Groupe Européen de Curiethérapie—European Society for Radiotherapy & Oncology) recommendations, with the CTV encompassing the whole uterus and the upper third of the vagina [14]. When MRI planning was available, a gross target volume (GTV) was defined as the residual tumor in T2-weighted sequences. Dose was prescribed to the CTV, and treatment plans were optimized to ensure adequate target coverage while respecting dose constraints to organs-at-risk (OARs), following GEC-ESTRO recommendations. When indicated, EBRT was delivered to the large pelvis using either a 3-D technique or volumetric arc therapy (VMAT). Target volumes were delineated according to institutional protocols consistent with international guidelines.

Clinical, radiological, pathological, and treatment-related variables were collected from electronic medical records. Baseline variables included age, clinical–radiological FIGO stage (2009), histological subtype and grade, molecular classification when available, BMI, and comorbidity burden assessed using the CCI. BMI was analyzed as a continuous variable and categorized according to standard definitions. Surgical complications were classified according to the Clavien–Dindo grading system. Surgical-related variables included type of primary treatment, surgical approach (laparoscopic, robotic-assisted, open, or vaginal), need for conversion, and performance of sentinel lymph node biopsy. For patients undergoing nodal assessment, the execution and success of sentinel lymph node mapping were recorded. Additional surgical variables included the use of lymphadenectomy and administration of adjuvant therapy. Radiotherapy-related variables included treatment modality (BT alone or combined with EBRT), total dose, fractionation schedule, and number of fractions. Technical aspects such as the type of applicator used, anesthesia during BT procedures, and imaging techniques employed for treatment planning (e.g., CT or MRI) were also collected.

The primary outcome was cancer-specific survival (CSS), defined as time from the end of treatment to death attributable to EC. Secondary outcomes included overall survival (OS), defined as time from the end of treatment to death from any cause; recurrence-free survival (RFS), defined as time from the end of treatment to recurrence; disease-free survival (DFS), defined as time from the end of treatment to recurrence or death by any cause; and treatment-related toxicity, measured by CTCAE criteria v. 5.0. Additionally, the type and timing of recurrence (local, locoregional, or distant), management of recurrence, and patient status at two and five years of follow-up were recorded as exploratory variables. The cause of death was recorded in all deceased patients.

Data was obtained through retrospective review of clinical records and analyzed using SPSS Statistics, version 29.0 (IBM Corp., Armonk, NY, USA). Continuous variables are presented as the mean ± standard deviation or median with interquartile range, as appropriate, and categorical variables as frequencies and percentages. Comparisons between groups were performed using the chi-square test or Fisher’s exact test for categorical variables and the Student’s *t*-test or Mann–Whitney U test for continuous variables, as appropriate. Survival outcomes were estimated using the Kaplan–Meier method and compared using the log-rank test. Hazard ratios (HRs) and corresponding 95% confidence intervals (CIs) were calculated to assess the effect of treatment on survival outcomes. A *p*-value < 0.05 was considered statistically significant.

## 3. Results

### 3.1. Tumor and Patient Characteristics

A total of 72 patients with clinical–radiological FIGO 2009 stage I–II EC were included, comprising 36 patients treated with definitive radiotherapy (BT+/− EBRT) and 36 matched patients treated with primary surgery. The two groups were well balanced in terms of baseline characteristics, including age, BMI, and comorbidity burden, with a mean age of 73.8 ± 9.8 years, a mean BMI of 38.1 ± 9.9 kg/m^2^, and a mean age-adjusted CCI of 6.9 ± 1.7. Endometrioid carcinoma was the predominant histological subtype. At diagnosis, most patients presented with early-stage disease (FIGO 2009 IA–IB). Molecular classification was retrieved when available; however, this information was not consistently accessible across both groups, particularly in the RT cohort. Detailed baseline characteristics are presented in Table 1.

### 3.2. Treatment Characteristics

Within the RT group, the main reasons for inoperability were comorbidities (56.8%), morbid obesity (30.6%), and anesthesia-related intraoperative complications (11.1%). Treatment included BT with or without EBRT. A total of 94.4% of patients completed the planned treatment. In the two patients who did not, the reasons were technical inability to perform BT due to inadequate uterine cavity size for applicator placement and early discontinuation of BT due to severe diarrhea, with only 15 of the initially planned 20 Gy delivered. All BT treatment plans were based on CT imaging with endovenous contrast. The most commonly used applicator was the exclusive double uterine tandem (72.2%, *n* = 26), followed by the single uterine tandem combined with a vaginal cylinder or ovoids (16.7% and 8.3%). The average doses administered, according to the RT techniques used, are detailed in Table 2.

In the surgical group, minimally invasive techniques were used in 69.4% of patients (58.3% robotic approach and 11.1% laparoscopic approach). Conversions to laparotomy occurred in 8% of cases, all due to uterine size. One intraoperative complication (2.8%) was recorded, consisting of acute hemorrhage requiring transfusion. In 52.8% of cases, lymph node evaluation was not performed as it was considered to provide limited benefit for patients with a high-risk of surgical morbidity.

Adjuvant treatment was indicated in 58.3% of patients (*n* = 21). Of these, seven received vaginal cuff BT alone, six received EBRT combined with BT, and seven received combined adjuvant treatment with chemotherapy + EBRT + BT.

### 3.3. Treatment-Related Complications

In the RT group, grade (G) ≥ 3 toxicity was observed in seven patients (19.4%), mainly involving gastrointestinal (G-I) and urinary events (11.6 and 7.8%, respectively). Specifically, three patients (8.3%) developed G3 urinary toxicity and three experienced G3 GI toxicity. In addition, one case of G4 rectal toxicity (2.8%) was recorded, corresponding to a rectovaginal fistula that ultimately required surgical management with definitive colostomy. No treatment-related mortality was observed.

Within the surgical group, three patients (8.3%) developed moderate-to-severe complications, including one postoperative death. Overall, no statistically significant differences were found in complication rates between groups (*p* = 0.22). Details on moderate-to-severe complications are provided in Table 3.

### 3.4. Survival Outcomes

The patient cohort was followed for a period of 5 years. The median follow-up time was 52.6 months and 53.2 months in the RT and surgery groups, respectively. At 4 months post-treatment, the complete radiological response rate was 71.4%, with partial responses in 25% of the cohort; 22.2% of patients were lost to follow-up.

**Figure 1:**
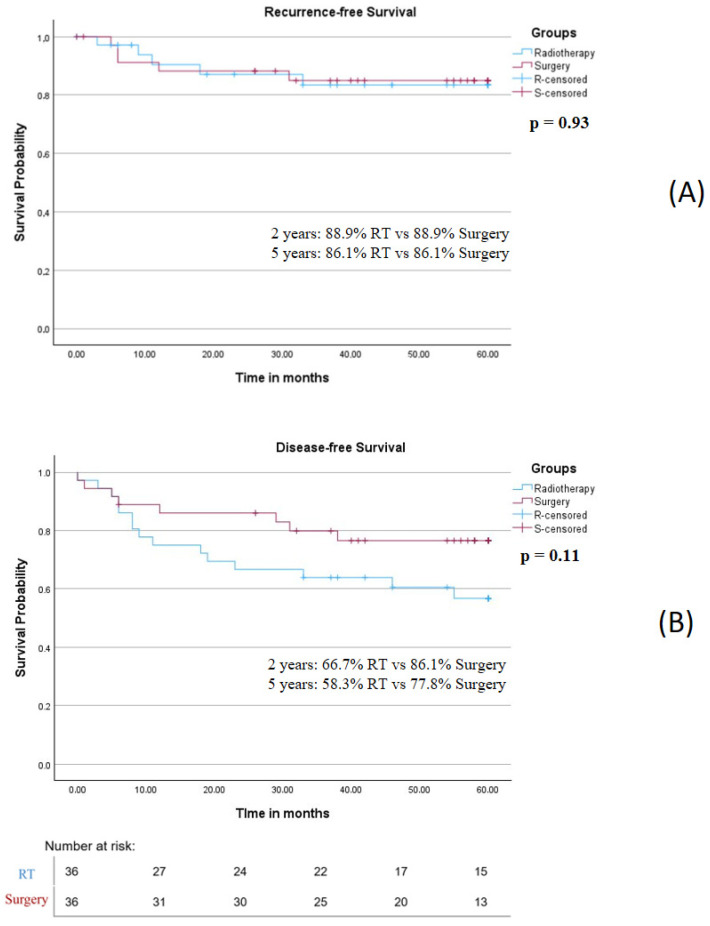
(**A**) Recurrence-free survival (RFS) at five years. (**B**) Disease-free survival (DFS) at five years. In this study, RFS and DFS were measured from treatment completion.

The 2 y overall recurrence rate was 11.1%, with an equal distribution between the two groups. At five years, the recurrence rate was 13.9% in both groups, corresponding to 5 cases per group (*p* = 1.0). In the RT group, recurrences included one case of residual disease, two locoregional recurrences, and two distant recurrences. In the surgery group, there were three locoregional recurrences and two distant recurrences. The median time to recurrence was 14.8 months in the RT group and 14 months in the surgery group. RFS and DFS curves are shown in Figure 1.

**Figure 2:**
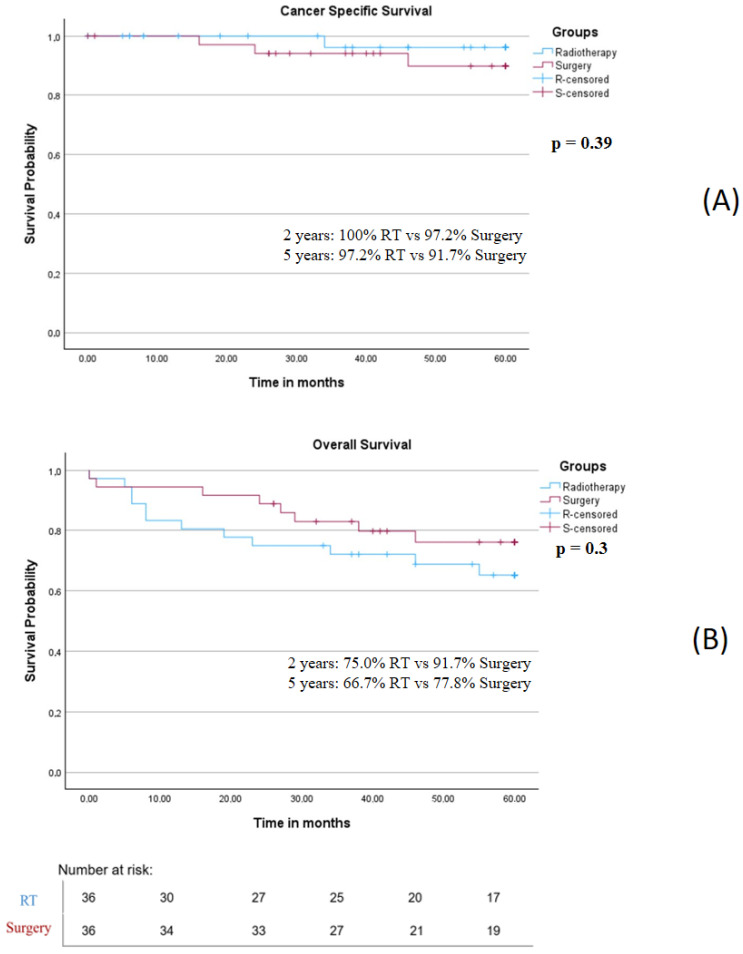
(**A**) Cancer-specific survival (CSS) at five years; (**B**) overall survival (OS) at five years. In this study, CSS and OS were measured from treatment completion.

At two years, one cancer-related death was observed in the surgery group. At five years, one patient in the RT group and three patients in the surgical group had died from EC, resulting in CSS rates of 97.2% and 91.7%, respectively (*p* = 0.39). For overall survival, nine deaths occurred in the RT group and three in the surgical group at two years (OS 75.0% vs. 91.7%, *p* = 0.06). At five years, twelve patients in the RT group and eight in the surgical group had died, with OS rates of 66.7% and 77.8%, respectively (*p* = 0.3). CCS and OS curves are shown in Figure 2. The characteristics of the 20 patients who died within the 5 y follow-up are detailed in Supplementary Table S1.

## 4. Discussion

The present study provides a direct comparison between definitive radiotherapy (RT) and primary surgery in a cohort of patients with early-stage EC, matched for age, BMI, and comorbidity burden. Our findings observe that RT achieves oncologic outcomes comparable to surgery in terms of CSS, DFS, and recurrence rates, despite being preferentially administered to high-risk surgical patients. This observation is clinically relevant as it challenges the traditional paradigm in which RT is considered merely an alternative for inoperable patients. Instead, our results support the concept that in appropriately selected high-risk populations, definitive RT represents a valid curative-intent strategy with equivalent cancer control.

The oncologic outcomes observed in the RT cohort were particularly encouraging. The 5-year CSS rate of 97.2% and recurrence rate of 13.9% are consistent with, and in some cases superior to, previously reported series of inoperable patients treated with brachytherapy-based approaches, where 5-year CSS rates typically range between 80% and 85% [12,13,15]. These results likely reflect both improvements in RT techniques and careful patient selection. In particular, the widespread use of BT enables precise target delineation and optimized dose delivery while minimizing exposure to organs at risk, contributing to local control while limiting exposure to surrounding organs [15,16,17].

Notably, patterns of recurrence differed between treatment groups, although they were not statistically significant (*p* = 0.55) (Table 4). In the RT cohort, most relapses were confined to the uterus or regional sites, whereas in the surgical group, a higher proportion of patients developed distant disease. This suggests that local control achieved with RT is effective and that recurrence patterns may be influenced by tumor biology [18]. In this regard, a significant imbalance in molecular classification was observed between groups (*p* < 0.01), which may have contributed to these differences.

However, these findings should be interpreted with caution as lymph node assessment was not performed in 53% of patients in the surgical group. This may have led to understaging and could partially explain the higher rate of nodal and distant recurrences observed in this cohort [19]. Despite this limitation, the surgical cohort achieved excellent oncologic outcomes, with a 5-year CSS of 91.7%, in line with previously published data [19,20,21].

Regarding treatment-related morbidity, some differences between the two approaches were observed. RT was associated with a moderate rate of grade ≥3 toxicity (19.4%), mainly gastrointestinal and urinary, but no treatment-related deaths were recorded. Although this rate appears slightly higher than reported in some series, it remains acceptable given the high-risk profile of the population [13,22,23]. Interestingly, this difference does not seem to be fully explained by dosimetric parameters alone. D2cc values were not consistently above commonly accepted thresholds, and severe toxicity was also observed in cases with apparently acceptable doses, suggesting that other factors may have contributed.

In this regard, technical aspects of treatment may be relevant. In our cohort, BT was not fully adaptive, MRI-based planning was not systematically used, and different EBRT techniques were applied [14,17,24]. In addition, intrafraction organ motion may have resulted in higher delivered doses than those estimated during planning. Overall, these findings suggest that dose volume parameters alone may not fully predict toxicity and highlight the importance of adaptive image-guided techniques and better control of organ motion to reduce complications.

Importantly, most RT-related toxicities were manageable and did not prevent treatment completion. In contrast, surgery was associated with a lower rate of severe complications, but included one perioperative death, underscoring the potential risks of surgery in this patient population [25,26,27]. These findings underline the importance of balancing oncologic outcomes with treatment-related morbidity, particularly in elderly and comorbid patients, where preserving functional status is a key consideration [1].

Although 5-year OS was lower in the RT group (66.7% vs. 77.8%), this difference was not statistically significant and was mainly driven by non-cancer-related mortality. This highlights the need to consider competing risks when making treatment decisions in this population [5,28]. The lower OS observed among patients receiving RT may reflect selection and referral biases. Specifically, these patients may have been referred from smaller centres without BT facilities or the capacity to perform complex surgery. In addition, patients with poorer prognostic profiles, greater comorbidity burden, or contraindications to surgery may have been preferentially referred to our BT reference unit.

Molecular classification is increasingly relevant for risk stratification and treatment selection in EC [28,29,30]. According to recent ESGO/ESTRO/ESP guidelines and the FIGO 2023 report, the integration of molecular subtypes (POLEmut, MMRd, NSMP, p53abn) refines prognostic assessment and may guide therapeutic strategies [5,30]. In our cohort, molecular data were incomplete—particularly in the RT group (61.1%)—limiting meaningful comparisons. Among available cases, NSMP tumors were the most frequent, and p53abn tumors were identified in a minority, as expected in early-stage disease. Although molecular classification is becoming increasingly important for risk stratification, its role could not be adequately explored in our series because of missing data. More complete molecular characterization in future studies may improve patient selection.

Advances in minimally invasive and robotic surgery have expanded the feasibility of surgery in selected obese patients. However, severe comorbidities still preclude surgery in some cases. In this setting, definitive RT remains a reasonable treatment for patients who are not suitable surgical candidates. These advances in surgery do not reduce the relevance of RT but rather highlight the need to select treatment according to each patient’s clinical condition.

It is important to note that patient selection in this study was largely based on negative criteria: patients received RT because surgery was not an option (primarily due to comorbidities, morbid obesity, or anaesthesia-related contraindications). This selection bias is inherent to the study design. In particular, the role of age deserves specific consideration. In our cohort, the mean age was 74 years in both groups, with no statistically significant difference. Importantly, advanced age per se was not a criterion for inoperability; rather, it was the accumulation of comorbidities—reflected by a mean age-adjusted CCI of 6.9—that determined surgical eligibility. This distinction is clinically relevant since age alone should not justify de-escalation of adjuvant therapy. Indeed, recent real-world data evaluating adjuvant external beam radiotherapy in elderly EC patients demonstrated that age was not a reason to withhold or reduce adjuvant treatment, with comparable tolerability profiles across age groups [31]. Our data are consistent with this perspective: treatment completion in the RT group was high (94.4%), supporting the feasibility and tolerability of curative-intent RT in elderly patients with EC when appropriately selected.

New treatment strategies are also being explored, including combinations of local treatment and systemic therapy [32,33]. These approaches may expand options for patients who are not candidates for surgery, but they are more likely to complement than replace RT within a multidisciplinary approach.

The results should be interpreted considering several limitations. First, the retrospective design, limited sample size, and incomplete availability of some variables inherently restrict the conclusions that can be drawn. Nevertheless, matching by BMI and comorbidity burden represents a relevant strength in this clinical setting. Second, there was considerable heterogeneity within the RT group regarding treatment techniques: patients received PDR brachytherapy, HDR brachytherapy, or EBRT alone, in various combinations, and with different external beam techniques (3-D conformal or VMAT), which may have introduced variability in outcomes within this group. Third, although a statistically significant difference in molecular profile distribution was observed between groups (*p* < 0.01), the very high rate of missing molecular data in the RT cohort (61.1% vs. 5.6% in the surgical group) precludes any firm conclusion regarding true between-group differences. Future studies should prioritise complete molecular profiling to allow adequate risk stratification and matched comparisons.

In addition, although RT dose may influence local control, we could not explore a possible dose–response relationship because of the low number of events. This remains an important area for future research. Likewise, the impact of weight-control interventions could not be assessed, although this remains relevant given the strong association between obesity and endometrial cancer [26].

In conclusion, definitive RT appears to be a safe and effective curative-intent option for patients with early-stage EC who are not candidates for surgery. Comparable oncologic outcomes and an acceptable toxicity profile support its role as a valid alternative to surgery in this population. Multidisciplinary evaluation remains essential, and future prospective studies integrating molecular classification and novel systemic therapies will be key to optimizing patient selection and improving outcomes.

## 5. Conclusions

Definitive BT, with or without EBRT, provides comparable oncological outcomes to surgery in patients with early-stage endometrial cancer (FIGO 2009 I–II) and high surgical risk. Although a trend toward higher complication rates was observed in the radiotherapy group, these events were predominantly manageable and may represent a more acceptable risk profile than surgery in frail and comorbid patients. These findings support definitive radiotherapy as a valid, radical alternative for carefully selected patients. Integration of molecular profiling may further refine patient selection and optimize personalized treatment strategies. Larger prospective trials are needed to confirm long-term outcomes.

## Figures and Tables

**Table 1:** Baseline characteristics of patients treated with radiotherapy or surgery. Data are presented as mean ± standard deviation or *n* (%).

	Radiotherapy (*n* = 36)	Surgery (*n* = 36)	*p*
Age (years ± standard deviation)	74.2 ± 9.9	73.4 ± 9.8	0.76
BMI (kg/m^2^)	37.8 ± 10.4	38.3 ± 9.4	0.82
Obesity (*n* (%))	27 (75%)	27 (75%)	1
Morbid obesity (*n* (%))	16 (44.4%)	17 (47.2%)	0.81
Charlson index (CCI) (value ± standard deviation)	7.1 ± 1.7	6.7 ± 1.6	0.36
CCI 0 (low)	0	0	
CCI 1–2 (moderate)	0	0	
CCI 3–4 (high)	2 (5.6%)	2 (5.6%)	
CCI ≥ 5 (very high)	34 (94.4%)	34 (94.4%)	
ASA PS (*n* (%))			0.19
II	8 (22.2%)	15 (41.6%)	
III	26 (72.2%)	20 (55.6%)	
IV	2 (5.6%)	1 (2%)	
Histology			0.30
Endometrioid	32 (88.8%)	32 (88.8%)	
Serous	0	1 (2.8%)	
Clear cell	0	2 (5.6%)	
Carcinosarcoma	1 (2.8%)	0	
Mixed	2 (5.6%)	0	
ADK arising in hyperplasia	1 (2.8%)	1 (2.8%)	
Histological grade			0.76
Hyperplasia	0	1 (2.8%)	
G1	19 (52.8%)	17 (47.2%)	
G2	8 (22.2%)	8 (22.2%)	
G3	9 (25%)	10 (27.8%)	
FIGO 2009 (at diagnosis)			0.95
Stage IA	18 (50%)	18 (50%)	
Stage IB	16 (44.4%)	15 (41.7%)	
Stage II	1 (2.8%)	2 (5.5%)	
NA	1 (2.8%)	1 (2.8%)	
Molecular profile			<0.01
POLE mutated	0	0	
Non-specific	8 (22.2%)	14 (38.9%)	
MSI	4 (11.1%)	17 (47.2%)	
p53 mutated	2 (5.6%)	3 (8.3%)	
NA	22 (61.1%)	2 (5.6%)	

BMI: body mass index; ASA: American Society of Anesthesiologists Physical Status; ADK: adenocarcinoma; MSI: microsatellite instability; NA: not available.

**Table 2:** Dosimetric parameters and treatment characteristics of brachytherapy +/− ERBT according to technique. Data are presented as median (range). Doses are expressed as equivalent dose in 2 Gy fractions (EQD2). D2cc corresponds to the dose to the most exposed 2 cm^3^ of each organ at risk.

	PDR (*n* = 4)	PDR + EBRT (*n* = 6)	HDR (*n* = 12)	HDR + EBRT (*n* = 13)	EBRT (*n* = 1)
CTV (cc)	105.9	101.0	86.0	90.5	-
D90 CTV (Gy)	60.7 (39.1–72.4)	67.9 (64.9–71.6)	44.5 (23.2–64.3)	69.1 (53.6–84.6)	-
Gy/Fx (Gy)	0.7–0.8	0.7–0.8	5.0–7.5	5.0–7.0	-
Number of Fx	71 (40–88)	30 (20–36)	7 (4–7)	3 (2–6)	-
D2cc organs at risk (Gy)					-
- D2cc rectum	24.6 (13.7–37.8)	56.8 (45–70.6)	26.7 (7.9–60.6)	51.8 (39.8–68.3)	
- D2cc bladder	80.8 (47.4–95)	67.9 (62.9–77.4)	43.9 (16.5–73)	65.0 (47.6–81.2)	
- D2cc sigma	65.8 (33.3–83.7)	53.9 (55–78.9)	38.1 (21.4–71.4)	60.3 (46.9–75)	
- D2cc bowels	52.0 (36.8–66.1)	49.4 (45.9–66.1)	37.0 (19.8–58.7)	53.2 (28–72.7)	
Dose EBRT (Gy)	-	46.7 (45–50.4)	-	43.4 (20–50)	50.4
EBRT planning volume		100% Large pelvis		84.6% large pelvis 15.4% uterus	100% Large pelvis
EBRT technique		33% 3-D 66.7% VMAT		46% 3-D 54% VMAT	3-D

PDR: pulse dose rate; HDR: high dose rate; D2cc: minimum dose received by the 2 cubic centimeters; Gy: gray; Fx: fraction; EBRT: external beam radiotherapy, 3-D: three-dimensional, VMAT: volumetric arc therapy.

**Table 3:** Detailed description of grade ≥ 3 complications according to treatment modality.

N°	Treatment	Complication	Grade CTCAE/C-D	Radiotherapy Scheme and Technique D2cc OAR (Gy)	Time (Months)	Management	Hospital Admission	Death
1	RT	Rectovaginal fistula	G4	HDR 7.5 Gy/fx 6 fx Rectum: 59.8	13	Colostomy	Yes	No
2	RT	Radiation cystitis + hematuria	G3	PDR 0.7 Gy/fx 36 fx + EBRT 3-D technique (45 Gy) Bladder: 77.4	32	Conservative	No	No
3	RT	Radiation cystitis + hematuria	G3	PDR 0.7 Gy/fx 33 fx + EBRT VMAT technique (45 Gy) Bladder: 70.7	15	UAE	Yes	No
4	RT	Radiation proctitis and cystitis + hematuria	G3	HDR 7 Gy/fx 3 fx + EBRT 3-D technique (only uterus, 40 Gy) Bladder: 60.1 Rectum: 61.6 Sigmoid: 58.1	28	Bladder irrigation	No	No
5	RT	Radiation proctitis and cystitis + hematuria	G3	PDR 0.8 Gy/fx, 31 fx + EBRT VMAT technique (45 Gy) Bladder: 67.1 Rectum: 70.6 Sigmoid:	22	5-ASA suppositories/corticosteroids	No	No
6	RT	Radiation proctosigmoiditis + lower GI bleeding	G3	HDR 5 Gy/fx 3 fx + EBRT 3-D technique (45 Gy) Rectum: 47.3 Sigmoid: 59.0	16	Transfusion	No	No
7	RT	Radiation proctosigmoiditis + lower GI bleeding	G3	HDR 7 Gy/fx 5 fx + EBRT 3-D technique (only uterus, 20 Gy) Rectum: 39.8 Sigmoid: 75	6	Transfusion	No	No
8	Surgery	Cardiac decompensation	V	-	2	Conservative	Yes	Yes
9	Surgery	Intra-abdominal collection and septic shock	IV	-	11	Antibiotic	Yes	No
10	Surgery	Wound dehiscence and abdominal wall necrosis	III	-	15	Surgery	Yes	No

G: grade; CTCAE: Common Terminology Criteria for Adverse Events; PDR: pulsed-dose-rate brachytherapy; HDR: high-dose-rate brachytherapy; EBRT: external beam radiotherapy; D2cc OAR: dose delivered to 2 cc of the organ at risk; UAE: uterine artery embolization; 5-ASA: 5-aminosalicylic acid; GI: gastrointestinal; C-D: Clavien–Dindo; 3-D: three-dimensional; VMAT: volumetric arc therapy; fx: fraction.

**Table 4:** Characteristics of patients with recurrence according to treatment modality.

Type of Relapse	Primary Treatment	Age (Years)	Histology	Grade	Molecular Profile	Stage FIGO 2009
isolated local (uterus)	RT	75	Endometrioid	3	p53abn	IB
local + pelvic peritoneum	RT	84	Mixed	1	NA	IB
local + pelvic nodes	RT	85	Endometrioid	1	MMRd	IA
distant metastases (umbilical and inguinal nodes)	RT	77	Carcinosarcoma	3	NA	IB
para-aortic nodes	RT	57	ADK arising in hyperplasia	1	NA	IA
local (vaginal dome)	Surgery	72	Endometrioid	2	MMRd	IA
pelvic implant	Surgery	85	Endometrioid	3	NSMP	IB
pelvic implant	Surgery	66	Endometrioid	3	MMRd	IB
distant metastases (lung)	Surgery	70	Endometrioid	2	MMRd	IA
pelvic + para-aortic nodes + distant metastases (liver)	Surgery	74	Serous	3	p53abn	IA

RT: radiotherapy; NA: not available; ADK: adenocarcinoma; NSMP: non-specific molecular profile; MMRd: mismatch repair defficient.

## Data Availability

The data underlying this study are not publicly available due to patient privacy and ethical restrictions. Data may be made available upon reasonable request to the corresponding author, subject to institutional approval.

## Supplementary Materials

The following supporting information can be downloaded at https://www.mdpi.com/article/10.3390/cancers18111858/s1, Table S1: Detailed characteristics of the 20 patients who died within 5 years of follow-up.

## Abbreviations

The following abbreviations are used in this manuscript:

ABS	American Brachytherapy Society
ASA PS	American Society of Anesthesiologists Physical Status
BMI	Body Mass Index
BT	Brachytherapy
CCI	Charlson Comorbidity Index
CI	Confidence Interval
CSS	Cancer-Specific Survival
CT	Computed Tomography
CTCAE	Common Terminology Criteria of Adverse Events
CTV	Clinical Target Volume
D2cc	Dose to the Most Exposed 2 cm^3^
DFS	Disease-Free Survival
EBRT	External Beam Radiotherapy
EC	Endometrial Cancer
EQD2	Equivalent Dose in 2 Gy Fractions
FIGO	International Federation of Gynecology and Obstetrics
G	Grade
GEC-ESTRO	Groupe Européen de Curithérapie—European Society for Radiotherapy & Oncology
GI	Gastrointestinal
GTV	Gross Tumor Volume
HDR	High Dose Rate
HR	Hazard Ratio
IGBT	Image-Guided Brachytherapy
IRB	Institutional Review Board
MRI	Magnetic Resonance Imaging
MSI	Microsatellite Instability
NA	Not Available
NSMP	No Specific Molecular Profile
OAR	Organ At Risk
OS	Overall Survival
PDR	Pulse Dose Rate
POLE	Polymerase Epsilon
RFS	Recurrence-Free Survival
RT	Radiotherapy
UAE	Uterine Artery Embolization
VMAT	Volumetric Modulated Arc Therapy
