# Supplementary material for: Primary Radiotherapy Versus Surgery in Early-Stage Endometrial Cancer Among High-Risk Surgical Patients: A Retrospective Comparative Study
Source: Cancers (Basel). 2026 Jun 5;18(11):1858. doi: 10.3390/cancers18111858 (PMC13256908; doi:10.3390/cancers18111858)
Supplement: Supplementary file 1 [file cancers-18-01858-s001.zip › cancers-4329384-supplementary.pdf]

Supplementary data

Supplementary Table S1. Detailed characteristics of the 20 patients who died within 5 years of follow-up.

Treatment	CSS	Cause of death	Age	CCI	2009 FIGO	Grade	Histology	Time (months)	Recurrence
RT	**No**	Disease	77	5	IB	G3	Carcinosarcoma	38	Local
RT	Yes	Decompensated CKD	80	11	IA	G2	Endometrioid	8	—
RT	Yes	Decompensated CKD	81	10	IB	G2	Endometrioid	9	—
RT	Yes	Decompensated CKD	88	9	IB	G1	Endometrioid	1	—
RT	Yes	Heart failure	83	6	IB	G1	Endometrioid	23	—
RT	Yes	Pyelonephritis with acute renal failure	67	9	IA	G1	Endometrioid	50	—
RT	Yes	Advanced liver cirrhosis and intestinal perforation	79	8	IA	G2	Endometrioid	11	—
RT	Yes	Decompensated liver cirrhosis	61	6	IA	G1	Endometrioid	9	—
RT	Yes	Decompensated liver cirrhosis	78	10	IA	G1	Mixed	55	—
RT	Yes	Cardiorespiratory arrest	82	7	II	G2	Endometrioid	26	—
RT	Yes	Cardiorespiratory arrest	85	9	IA	G1	Endometrioid	24	Locoregional
RT	Yes	Cardiorespiratory arrest	96	7	IB	G3	Endometrioid	10	—
Surgery	**No**	Disease	70	7	IA	G2	Endometrioid	46	Distant
Surgery	**No**	Disease	74	7	IIIC2	G3	Mixed	24	Distant
Surgery	**No**	Disease	85	7	IIIA	G2 / G3	Endometrioid	28	Local
Surgery	Yes	Septic shock	70	10	IIIA	G1	Endometrioid	42	—
Surgery	Yes	Cardiorespiratory arrest	87	10	II	G3	Clear cell	0.3 (10 days)	—
Surgery	Yes	Acute myocardial infarction	71	5	IB	G2	Endometrioid	2	—
Surgery	Yes	Cerebrovascular accident	84	8	IA	G2	Endometrioid	32	—
Surgery	Yes	Amyotrophic lateral sclerosis	66	5	IB	G2	Endometrioid	31	—

CSS: cancer-specific survival; RT: radiotherapy; FIGO: International Federation of Gynecology and Obstetrics staging system (2009); G: grade. CKD: chronic kidney disease; CCI: Charlson Comorbidity Index.
